# Multicomponent mHealth Intervention for Large, Sustained Change in Multiple Diet and Activity Risk Behaviors: The Make Better Choices 2 Randomized Controlled Trial

**DOI:** 10.2196/10528

**Published:** 2018-06-19

**Authors:** Bonnie Spring, Christine Pellegrini, H G McFadden, Angela Fidler Pfammatter, Tammy K Stump, Juned Siddique, Abby C King, Donald Hedeker

**Affiliations:** ^1^ Department of Preventive Medicine Feinberg School of Medicine, Northwestern University Chicago, IL United States; ^2^ Health Research and Policy Stanford University School of Medicine Stanford, CA United States; ^3^ Department of Public Health Sciences The University of Chicago Chicago, IL United States

**Keywords:** health behavior, risk factors, mobile health, behavioral medicine, randomized controlled trial

## Abstract

**Background:**

Prevalent co-occurring poor diet and physical inactivity convey chronic disease risk to the population. Large magnitude behavior change can improve behaviors to recommended levels, but multiple behavior change interventions produce small, poorly maintained effects.

**Objective:**

The Make Better Choices 2 trial tested whether a multicomponent intervention integrating mHealth, modest incentives, and remote coaching could sustainably improve diet and activity.

**Methods:**

Between 2012 and 2014, the 9-month randomized controlled trial enrolled 212 Chicago area adults with low fruit and vegetable and high saturated fat intakes, low moderate to vigorous physical activity (MVPA) and high sedentary leisure screen time. Participants were recruited by advertisements to an open-access website, screened, and randomly assigned to either of two active interventions targeting MVPA simultaneously with, or sequentially after other diet and activity targets (N=84 per intervention) or a stress and sleep contact control intervention (N=44). They used a smartphone app and accelerometer to track targeted behaviors and received personalized remote coaching from trained paraprofessionals. Perfect behavioral adherence was rewarded with an incentive of US $5 per week for 12 weeks. Diet and activity behaviors were measured at baseline, 3, 6, and 9 months; primary outcome was 9-month diet and activity composite improvement.

**Results:**

Both simultaneous and sequential interventions produced large, sustained improvements exceeding control (*P*<.001), and brought all diet and activity behaviors to guideline levels. At 9 months, the interventions increased fruits and vegetables by 6.5 servings per day (95% CI 6.1-6.8), increased MVPA by 24.7 minutes per day (95% CI 20.0-29.5), decreased sedentary leisure by 170.5 minutes per day (95% CI –183.5 to –157.5), and decreased saturated fat intake by 3.6% (95% CI –4.1 to –3.1). Retention through 9-month follow-up was 82.1%. Self-monitoring decreased from 96.3% of days at baseline to 72.3% at 3 months, 63.5% at 6 months, and 54.6% at 9 months (*P*<.001). Neither attrition nor decline in self-monitoring differed across intervention groups.

**Conclusions:**

Multicomponent mHealth diet and activity intervention involving connected coaching and modest initial performance incentives holds potential to reduce chronic disease risk.

**Trial Registration:**

ClinicalTrials.gov NCT01249989; https://clinicaltrials.gov/ct2/show/NCT01249989 (Archived by WebCite at https://clinicaltrials.gov/ct2/show/NCT01249989).

## Introduction

Public health advocates have long endorsed an approach that prioritizes achieving small risk reductions for most of the population over large improvements for the minority of the population who are at high risk [1-5]. A middle-road between the population and high-risk approaches is emerging from the realization that much of the population lacking biologic cardiometabolic risk factors is not truly at low risk [6,7]; instead, they manifest equally impactful behavioral risk factors that warrant targeting for primordial prevention of disease [8-12].

The average adult reports at least two chronic disease risk behaviors; 25% report three or more; and the magnitude of behavior change needed to bring each risk factor into compliance with public health guidance is typically large [13-16]. Unhealthy diet and activity behaviors are the most prevalent lifestyle risks. Fewer than 15% of US adults eat five or more servings of fruits and vegetables daily; median intake is about half that amount [17]. Only 29% meet dietary guidelines to consume less than 10% calories from saturated fats [18-20]. Half fall short of public health recommendations for moderate to vigorous physical activity (MVPA) [21,22], and more than 50% exceed two hours per day watching television [21,23]. These four behaviors associate separately with heightened risk of cardiovascular disease and cancers, and link with others that predict premature mortality [9,10,24-30].

To date, the diet and activity changes produced by most multiple behavior change interventions have been small and poorly maintained. Conversely, when interventions have produced large initial behavior changes, long term effectiveness has been greater. However, skepticism persists about whether individuals without disease can be motivated to make and maintain large behavioral changes. We hypothesize that community dwelling adults with multiple diet and activity risk behaviors could be activated to achieve and maintain guideline levels of these behaviors by a scalable, multicomponent intervention that integrates mHealth technology, modest incentives, and remote connected coaching.

Inclusion of intervention components was guided by three principles—effectiveness, scalability, and synergy. Telephone coaching was used because the approach has demonstrated effectiveness and greater reach than in-person counseling [31,32]. On the other hand, although remote coaching is more scalable than in-person treatment, it produces smaller behavior changes [32]. Because larger magnitude behavior changes are maintained better [33], modest incentives were used that would maximize initial behavior changes [34,35]. In a prior study, we observed that incentives motivated participants to make changes of greater magnitude than they thought they could accomplish, and after being successful, most tried to maintain gains [36]. A smartphone app and accelerometer were used to provide diet and physical activity feedback synchronously to participants and their coaches, enabling connected, maximally personalized, adaptive coaching.

The Make Better Choices 2 (MBC2) trial aimed to improve upon the most effective intervention from a previous (MBC1) trial that also treated adults with four concurrent diet and activity risk behaviors: low fruit and vegetable intakes, high saturated fat intake, low MVPA, and high sedentary leisure screen time [36]. MBC1 results showed that participants incentivized for 3 weeks to increase fruits and vegetables, while decreasing leisure screen time made large sustained improvements in targeted behaviors as well as in saturated fat, which was untargeted. However, MVPA did not improve. Hence, the MBC2 trial tested whether also targeting MVPA simultaneously with or sequentially after the other diet and activity behaviors could optimize all four diet and activity behaviors, relative to a contact-control intervention.

## Methods

### Study Design, Population, and Procedures

The MBC2 study [37], was a three-arm prospective randomized controlled trial which compared two sequences of diet and activity intervention to a contact-control intervention. The active interventions targeted MVPA either simultaneously with (simultaneous) or sequentially after (sequential) other diet and activity risk behaviors (fruits and vegetables, sedentary leisure screen time). Saturated fat was not targeted explicitly because findings from the previous MBC1 trial showed that increasing fruits and vegetables and decreasing leisure screen time automatically lowered fat intake, as a tag-along healthy lifestyle improvement. The reduction in fat intake occurred effortlessly (without explicit goal setting), as increased fiber intake crowded out fat intake and as decreased screen time reduced the snacking with which it was usually paired [36]. Because prior findings show that trying to accomplish too many behavior changes simultaneously can be detrimental [38-40], we reduced participant burden by not setting fat goals, or providing app feedback for fat intake, or coaching about fat. Rather, the MBC2 intervention aimed to lower fat intake incidentally by increasing substitute behaviors (fruit and vegetable intake) that could crowd it out and by decreasing complementary behaviors (leisure screen time) that could cue it.

The control intervention addressed stress and sleep. Eligible participants were stratified by gender and individually randomized to a condition using randomly permuted blocks with an allocation ratio of 2:2:1 (simultaneous: sequential: control).

Adults, recruited through subway, bus, flyers, and newspaper advertisements, were referred to a website to complete online screening. Inclusion criteria were willingness to be randomized, age 18-65 years, and meeting all the following: <5 servings of fruits and vegetables per day; ≥8% daily calories from saturated fat; <150 minutes per week MVPA; >120 minutes per week of leisure screen time (ie, television, movies, videogames, recreational internet). These discretionary activities were targeted because they can be decreased without jeopardizing necessary work-related activities. Exclusion criteria were unstable medical condition (ie, uncontrolled hypertension or diabetes), pregnancy or intent to become pregnant, anorexia, bulimia, binge eating disorder, or weight >350 lb [37].

The Northwestern University Institutional Review Board approved all procedures, and the study was conducted in Chicago between July 2012 and July 2014 (see Multimedia Appendix 2 for trial protocol). After screening, eligible candidates attended an in-person session where they discussed the pros and cons of the three treatment options, provided written informed consent, and were loaned a smartphone and accelerometer. They were trained to estimate portion sizes, use the assessment version of a custom-built smartphone app to record behaviors (dietary intake, leisure screen time, stress level, relaxation exercises, and sleep), and wear an accelerometer for a baseline week. Instructions emphasized entering all meals and snacks immediately after eating and using sliders to show accumulated leisure screen time four times daily. Study apps processed dietary data through the integrated CalorieKing food database with all fruits and vegetable items tagged as serving sizes [41]. The Shimmer accelerometer [42], worn in a spibelt around the waist, recorded activity counts and wirelessly transmitted data through Bluetooth to the app, which converted counts to MVPA minutes. Following the in-person session, all eligible participants were sent home with a smartphone, app, and accelerometer to monitor themselves for seven to ten days. The self-monitored data were then assessed at the end of the baseline period and those who exhibited all four diet and activity risk behaviors throughout baseline were randomized.

**Figure 1:**
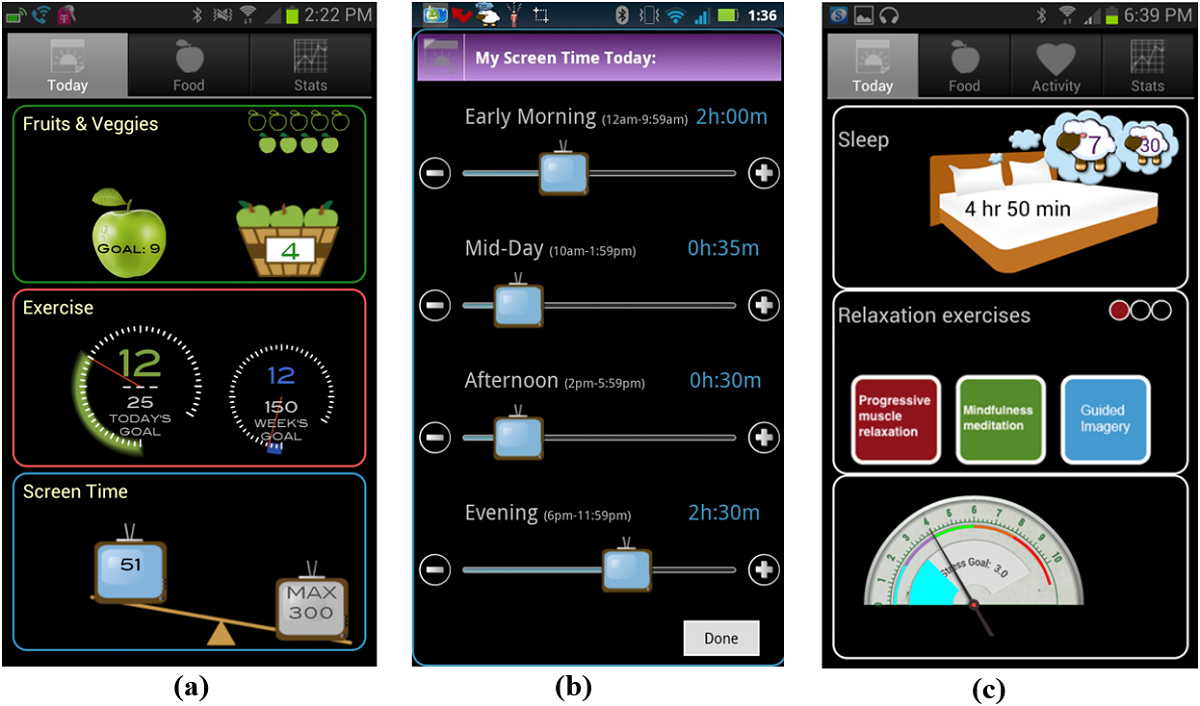
Make Better Choices 2 app user interfaces for (a) receiving behavioral feedback in simultaneous and sequential treatments; (b) reporting sedentary leisure screen time in simultaneous and sequential treatments; (c) receiving feedback in contact control treatment.

Participants learned their treatment assignment by downloading one of three custom-built, intervention-specific study apps [37]. They were asked to use the app and accelerometer to continuously record those behaviors the intervention targeted (ie, dietary intake, sedentary leisure, and MVPA for the aimultaneous and aequential conditions; stress, relaxation exercises, and sleep for control) throughout the 9-month study period. Unlike the assessment app, which collected user self-reports and accelerometer data but gave no user feedback, intervention apps provided users with continuously updated feedback about their performance of targeted behaviors relative to goal. The app user interfaces are shown in Figure 1. In addition to giving participants goal attainment feedback for targeted behaviors, apps wirelessly transmitted this information to coaches, who used it to tailor telephone counseling. Sequential and simultaneous apps were similar, except that the physical activity interface for sequential treatment remained inactive until week seven. End goals for the 12-week simultaneous or sequential intervention were: 1) ≤90 min per day of sedentary leisure screen time; 2) ≥5 servings of fruits and vegetables; and 3) ≥150 min per week of MVPA. Those receiving simultaneous treatment were asked to gradually modify all three target behaviors from the outset of the intervention. Those receiving sequential treatment were asked to modify only sedentary leisure screen time and fruit and vegetables for the first 6 weeks. Between weeks 7 and 12, they were asked to maintain goal levels for leisure screen time and fruit and vegetables, while progressively increasing MVPA. Control participants were coached to perform three relaxation exercises per day (a progressive muscle relaxation technique, a mindfulness meditation, and a self-hypnosis technique) [43], and to achieve end goals of ≥7.5 hours of sleep per day and a 30% reduction in stress over the 12-week intervention. Participants used a 1 to 10 Subjective Units of Distress Scale (SUDS) to record stress, as shown in Figure 1.

**Figure 2:**
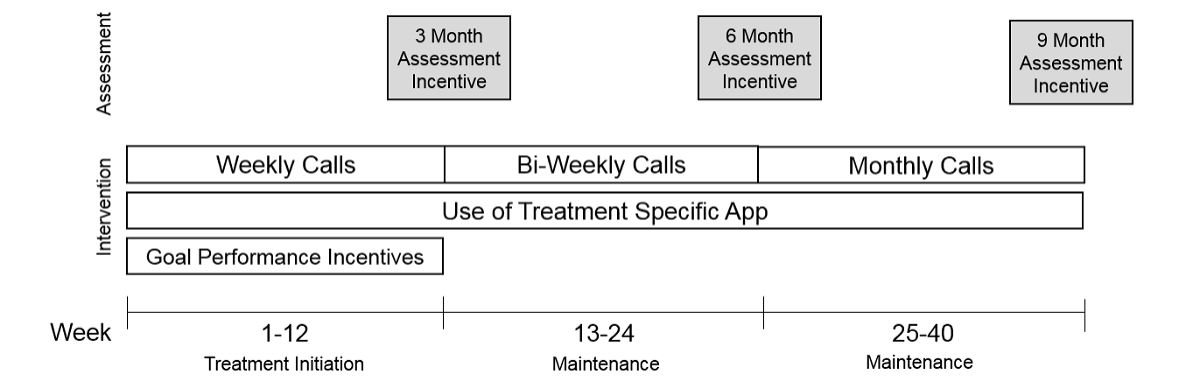
Make Better Choices 2 Trial study timeline.

During treatment initiation (weeks 1-12), a trained paraprofessional telephoned each participant weekly for a 15 minute coaching session. Coaches delivered a sequence of online didactic lessons specific to each condition [37] and used motivational interviewing to tailor counseling using data from the participant’s app and accelerometer. Coaching call frequency decreased to biweekly in weeks 13-24 and monthly in weeks 25-40, and call duration decreased to 10 minutes. The study timeline is shown in Figure 2. All coaching sessions were audiotaped; a 10% random sample selected quarterly was coded for treatment fidelity by blinded raters. Coaches scoring less than 90% were retrained and certified before making additional calls.

### Study Outcomes and Statistical Analysis

The primary trial outcome was the composite diet and activity improvement score measured for 1-week assessment periods at 3, 6, and 9 months when participants wore an accelerometer and used the assessment app to self-monitor their behaviors without receiving any feedback. Secondary outcomes were healthy changes in fruits and vegetables, saturated fat, MVPA, and leisure screen time. Measuring diet and activity by recording on a mobile device has shown acceptable reliability and validity and greater adherence compared to paper reporting or recall [44-46].

Changes in fruit and vegetable intakes, saturated fat intake, sedentary leisure screen time (derived from app self-report), and MVPA (derived from accelerometry supplemented by app self-report in the event of water activities or battery failure) were aggregated to calculate the MBC composite diet and activity improvement score, weighting each behavior equally [36]. Square root transformation was applied to count data (fruit and vegetables, MVPA, sedentary leisure screen time) and arcsine transformation was applied to the percentage outcome (fat) to better approximate normality.

Behaviors were placed on a common scale by standardizing each one using a modified Z*-* score (where one U represents a one-SD change) based on the baseline sample means and standard deviations of each behavior; higher values represent greater healthy lifestyle improvement relative to the overall baseline distribution. The MBC composite diet and activity improvement score [36], expressing each participant’s overall healthy change across the multiple diet and activity behaviors relative to baseline is calculated as the mean of all four individual Z scores at each time point.

Three components of treatment implementation were assessed: fidelity, receipt, and adherence [47]. Fidelity was considered present for sessions when the coach delivered all required treatment elements correctly (eg, encouraging a control participant to go to bed earlier) and absent if the coach delivered any incorrect treatment element (eg, encouraging a control participant to exercise to be tired at bedtime). Treatment receipt was measured by the percent of completed scheduled coaching calls. Self-monitoring adherence was assessed by the proportion of days that participant used the app to record targeted behaviors for 1-week assessments at baseline, 3, 6, and 9 months. Goal attainment was assessed by the proportion of weeks during treatment initiation when the participant met behavioral goals. To be credited with having met goal and to earn an incentive for the week, participants’ average scores for all the three target behaviors needed to meet or exceed goal level.

The average effect size (mean difference in composite diet and activity improvement Z-score divided by common standard deviation) in our previous MBC1 trial equaled 0.46. Based on power calculations, we aimed to recruit 50 control subjects and 100 subjects into each of the two intervention groups, assuming a correlation of 0.50 for the composite Z-scores across time and an attrition rate of 20% at the final time point. We powered the study for an effect size in the range of 0.5 for the first Helmert contrast (H1: simultaneous + sequential vs control) and 0.4 for the second Helmert contrast (H2: simultaneous vs sequential). Bankruptcy of the mobile phone service provider necessitated the return and provision of new mobile phones, creating a budget shortfall that required reducing enrollment from 250 to 212 participants: 84 allocated to the simultaneous intervention; 84 allocated to sequential; and 44 to control. However, because the observed correlation of the composite Z-scores over time was smaller (*r*=0.44) than the predicted 0.50, the study remained sufficiently powered for the posited effect sizes.

Baseline characteristics were compared across groups using analysis of variance (ANOVA) for continuous variables and chi-squared tests for categorical variables. The percentage of coaching calls received and completed in the first versus the second 6 weeks of treatment was analyzed using repeated measures ANOVA. The percentage of days participants adhered to self-monitoring was measured at baseline, 3-, 6-, and 9-months and analyzed using repeated measures ANOVA. Goal attainment (yes or no), measured every 2 weeks during treatment initiation, was analyzed using mixed-effects logistic regression with time modeled as either the first or the second 6-weeks of initiation. All models of treatment receipt, adherence, and goal attainment included group by time interactions to assess differences between groups at each time point.

Intent-to-treat analyses of primary and secondary endpoints used three-level linear mixed-effects models that treated daily measurements (level one) nested within 1-week assessment periods (level two) nested within subjects (level three). Thus, we analyzed at the daily level and considered the correlation of the daily measurements within weeks and subjects by including random subject intercept and time trends at level three (subjects), and a random intercept at level two (1-week assessment periods). For comparisons across assessment periods, we treated baseline as the reference, and estimated changes at 3-, 6-, and 9-month follow-up. For comparisons between the intervention groups Helmert contrasts were used, in which the first contrast compared the combined simultaneous and sequential groups to control, and the second contrast compared the simultaneous to the sequential group. We also included group by time interactions to assess the degree to which change from baseline varied for either of the Helmert contrasts at each follow-up.

## Results

Participants were primarily female (162/212, 76.4%), minority (125/212, 59.0%), college educated (147/212, 69.3%), with a mean age of 40.8 (11.9) years and mean BMI 34.3 (8.8). The groups did not differ in their baseline characteristics (Table 1).

**Figure 3:**
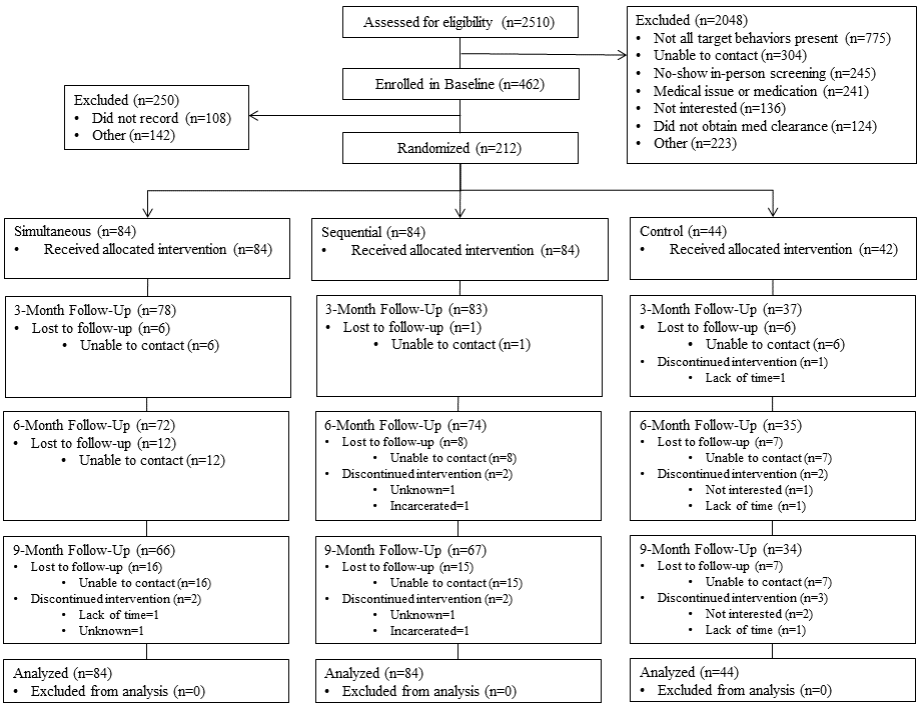
CONSORT Flow Diagram of the Make Better Choices 2 Trial: 212 adults were eligible for inclusion, randomly assigned to an intervention condition, and included in analyses.

Of study applicants who were web-screened, 18.4% proceeded through in-person screening and on into baseline recording to verify the presence of all four risk behaviors. Most of those listed in Figure 3 as excluded for “Other” reasons only partially completed the web screener. Of candidates who underwent baseline screening, 45.9% (212/462) were randomized. Two candidates randomly assigned to the stress and sleep contact control condition failed to receive the allocated intervention: both withdrew before receiving any treatment.

Loss to follow-up was 17.9% (38/212) and not differential across treatments (Figure 3). In the composite Z analysis, 83.5% (177/212) of participants provided a composite Z-score at two or more time points; 68.4% (145/212) provided three or more; and 50.5% (107/212) provided all four time points.

**Figure 4:**
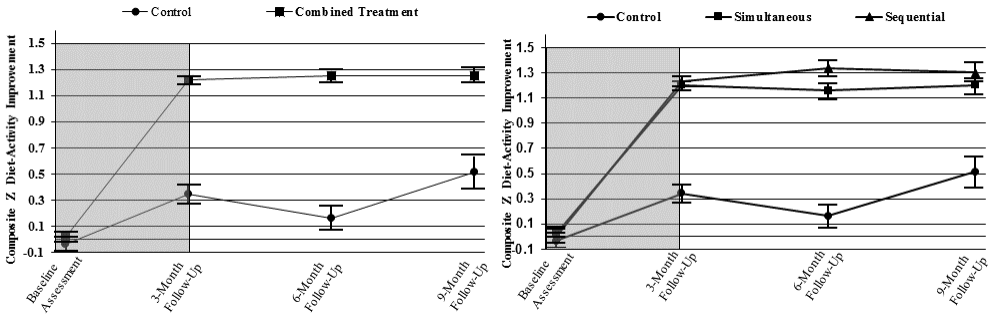
A: Mean Composite Diet-Activity Improvement Scores over time for combined simultaneous and sequential treatment groups vs control. Combined treatment groups produced greater healthy change at each postbaseline assessment point. B: Mean Composite Diet-Activity Improvement Scores over time for each of the three conditions. Error bars represent 1 SE. Gray background indicates the treatment initiation phase (weeks 0-12); white background, follow-up maintenance phase.

The combined simultaneous and sequential interventions produced sustained improvement, as compared to control, on the composite diet and activity score at 3, 6, and 9 months (*P*<.001; see Table 2 and Figure 4 A). Sequential treatment produced a small, significantly greater composite diet and activity improvement than simultaneous treatment at 6 months (*P*=.03); however, no differences were evident at 3 and 9 months (see Table 2 and Figure 4 B).

**Table 1:** Participant Baseline Characteristics.

Variable		Total (n=212)	Control (n=44)	Simultaneous (n=84)	Sequential (n=84)	Treatment group differences	
						Test	*P* value
Age (years), mean (SD)		40.8 (11.9)	40.8 (10.9)	40.7 (11.9)	40.9 (12.5)	F=0.003	.99
Body mass index (kg/m^2^), mean (SD)		34.3 (8.8)	36.0(10.1)	33.7(9.0)	33.9(7.9)	F=1.07	.34
**Gender, n (%)**							
	Male	50 (23.6)	11 (25.0)	20 (23.8)	19 (22.6)		
	Female	162 (76.4)	33 (75.0)	64 (76.2)	65 (77.4)	χ^2^=0.95	.95
**Race, n (%)**							
	Caucasian	87 (41.0)	19 (43.2)	28 (33.3)	40 (47.6)		
	Black	99 (46.7)	19 (43.2)	42 (50.0)	38 (45.2)		
	Asian	8 (3.8)	2 (4.5)	5 (6.0)	1 (1.2)		
	Other or multiple	18 (8.5)	4 (9.1)	9 (10.7)	5 (6.0)	χ^2^=9.28	.32
**Ethnicity, n (%)**							
	Hispanic/Latino	20 (9.8)	6 (14.0)	5 (6.3)	9 (11.0)		
	Not Hispanic/Latino	184 (90.2)	37 (86.0)	74 (93.7)	73 (89.0)	χ^2^=2.04	.36
**Education, n (%)**							
	College degree	147 (69.3)	32 (72.7)	60 (71.4)	55 (65.5)		
	No college degree	65 (30.7)	12 (27.3)	24 (28.6)	29 (34.5)	χ^2^=1.00	.61

**Figure 5:**
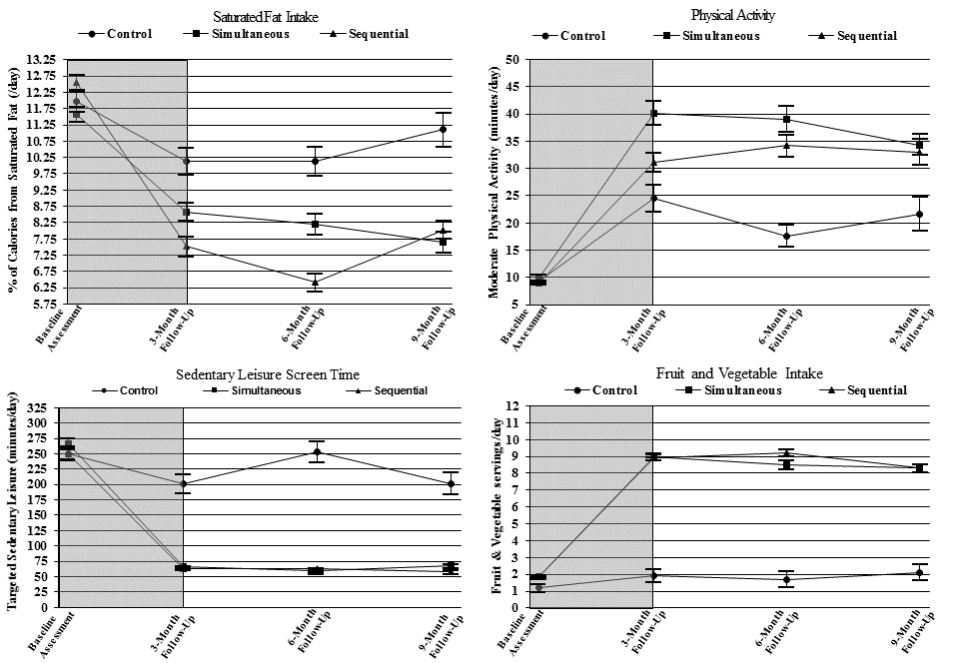
Effects of the 3 intervention conditions on changes over time in each behavior expressed in natural units. Gray background indicates treatment initiation phase (weeks 0-12); white background, follow-up maintenance phase.

Figures 4 and 5 show the behavior changes over time of each treatment group. Both simultaneous and sequential interventions improved each of the four behaviors to guideline levels, exceeding control at all three follow-up assessments (See Table 2). At 9 months, intervention increased the two healthful behaviors: (1) fruit and vegetable intake by 6.5 servings per day (95% CI 6.1-6.8), mean difference from control=6.2 servings per day (95% CI 5.5-6.9), and (2) MVPA by 24.7 min per day (95% CI 20.0-29.5), mean difference from control=12.1 min per day (95% CI 5.4-18.9); and decreased the two unhealthful behaviors: (1) sedentary leisure –170.5 min per day (95% CI –183.5 to –157.5), mean difference from control=–137.7 min per day (95% CI –155.9 to –119.5), and (2) saturated fat intake –3.6% (95% CI –4.1 to –3.1), mean difference from control=–3.3% (95% CI –4.3 to –2.2).

In addition to being sustained, the improvements produced by the active interventions were generally large (0.5 to 1.7 standard deviation unit improvements compared to baseline). Although Sequential treatment reduced saturated fat intake more than simultaneous treatment at 3 months (mean difference –1.1%, 95% CI –1.8 to –0.3) and 6 months (mean difference –1.8%, 95% CI –2.6-1.0, no difference was evident at 9 months, and no other differences between simultaneous and sequential treatments were observed (Table 2).

Treatment fidelity averaged 96.8% across the 2-year study; 3 out of 20 coaches required retraining. Receipt of calls declined from 66.0% during the first half of treatment initiation to 57.7% during the second half (F[1209]=12.05, *P*<.001), not differing among treatment groups (61.9%, 95% CI 58.0-65.7; *P*=.12). Self-monitoring decreased from an average of 96.3% at baseline to 72.3% at 3 months, 63.5% at 6 months, and 54.6% at 9 months (F[3627]=95.0, *P*<.001), without differences across intervention groups (*P*=.41). Goal attainment was greater for the Active intervention groups (58.8%, 95% CI 52.2% to 65.0%) than control (33.6%, 95% CI 23.1% to 46.0%) during the first half of treatment initiation, (*z*=3.46, *P*<.001), but Active and Control groups did not differ during the last half of treatment (38.3%, 95% CI 32.6% to 44.2%; *z*=0.13, *P*=.89).

**Table 2:** Differences in Standardized (Z-score) Change from baseline between treatments at follow-up. Italics indicate statistical significance.

Behavioral outcomes			3-Month follow-up, mean (95% CI)		6-Month follow-up, mean (95% CI)		9-Month follow-up, mean (95% CI)
**Combined vs control**							
	Sedentary Leisure Screen Time	*0.94 (0.65-1.23)* ^a^		*1.30 (0.95-1.64)* ^a^		*0.85 (0.42-1.28)* ^a^	
	Physical Activity	*0.66 (0.40-0.91)* ^a^		*0.86 (0.56-1.20)* ^a^		*0.54 (0.17-0.91)* ^b^	
	Fruit & Vegetable Intake	*1.74 (1.50-1.98)* ^a^		*1.72 (1.43-2.00)* ^a^		*1.37 (1.02-1.71)* ^a^	
	Saturated Fat Intake	*0.45 (0.19-0.72)* ^a^		*0.57 (0.27-0.87)* ^a^		*0.66 (0.32-0.99* ^a^	
	Composite Diet-Activity Score	*0.95 (0.80-1.10)* ^a^		*1.16 (0.98-1.34)* ^a^		*0.92 (0.69-1.14)* ^a^	
**Simultaneous vs sequential**							
	Sedentary Leisure Screen Time	–0.10 (–0.34-0.14)		–0.04 (–0.33-0.25)		0.00 (–0.36-0.36)	
	Physical Activity	–0.14 (–0.36-0.09)		0.02 (–0.24-0.28)		0.02 (–0.30-0.34)	
	Fruit & Vegetable Intake	–0.06 (–0.26-0.15)		0.10 (–0.14-0.34)		–0.05 (–0.35-0.24)	
	Saturated Fat Intake	*0.37 (0.14-0.60)* ^a^		*0.50 (0.24-0.75)* ^a^		0.15 (–0.13-0.44)	
	Composite Diet-Activity Score	–0.01 (–0.13-0.12)		*0.16 (0.01-0.31)* ^c^		0.06 (–0.13-0.25)	

^a^*P*<.001

^b^*P*<.01

^c^*P*<.05

## Discussion

These findings support the hypothesis that adults with multiple diet and activity risk behaviors can be activated to make and maintain large improvements in diet and activity behaviors by a scalable, multicomponent intervention that integrates mHealth technology, modest incentives, and remote-connected coaching by trained paraprofessionals. Both active MBC2 interventions produced larger sustained improvements in fruit and vegetables, saturated fat, physical activity, and sedentary leisure screen time than those observed in most prior trials, including our MBC1 study [13,36,48-51], such that all four behaviors surpassed guideline recommended levels at the final study follow-up. Also, unlike the prior MBC1 interventions, both active MBC2 interventions yielded a sustained increase in MVPA documented by accelerometry [51,52]. We attribute the greater maintenance of positive diet and activity changes in the MBC2 study to the longer intervention (12 weeks versus three) and continued availability of intervention technology with some minimal coach contact.

To our knowledge, the present study is unique in testing simultaneous vs sequential versions of a multicomponent mHealth intervention to address the four most prevalent, co-occurring diet and activity risk behaviors [38,39,49,53-56]. The findings show comparable improvement from intervening to increase MVPA simultaneously with or sequentially after targeting other diet and activity risk behaviors. Even though sequential treatment produced somewhat greater improvement than Simultaneous treatment in healthy diet and activity at 6 months, the difference was small in magnitude and not sustained. These findings expand to four the number of co-occurring poor diet and activity habits that can demonstrably be changed simultaneously and support the conclusion that either a simultaneous or a sequential approach to multiple health behavior change can be expected to yield benefit [54,55].

The unusually large, well-maintained diet and activity improvements observed in this trial are likely attributable to the effectiveness of the MBC2 intervention components, including the use of appealing mHealth technology and connective coaching as vehicles to deploy effective behavior change techniques (goal-setting, self-monitoring, feedback, support, accountability). Unlike recent trials that provided digital feedback solely to patients and failed to find benefit from supplying a wearable accelerometer [57,58], MBC2 provided feedback synchronously to both participants and coaches, enabling synergistic benefit through connected counseling. Strong engagement with the intervention and study technology resulted, evidenced by participants self-monitoring on 50% of days even after 9 months.

The MBC2 study’s US $5 per week incentive for participants to meet behavioral goals during treatment initiation apparently had the intended effect of motivating participants to make large improvements. Notably, MBC2 participants made somewhat larger diet and activity improvements than those in the MBC1 study, even though the MBC2 incentive was two-thirds smaller. Moreover, no incentive to sustain healthful diet and activity changes was operative in either the MBC1 or MBC2 trial; nevertheless, behavioral improvements were maintained. Hence, these findings contradict the worry that use of incentives followed by their discontinuation inevitably undermines behavioral maintenance. Results accord with a growing body of evidence showing sustained improvements after incentives cease [34,35]. Potential scalability of modest incentives is suggested by Centers for Medicare and Medicaid reimbursement of contingency contracting for some habit disorders, and by the growing number of individuals and employers that find incentives for healthy lifestyle change cost effective [59].

Strengths of the study include a strong scientific premise grounded in the high population prevalence of individuals whose multiple diet and activity risk behaviors place them at moderately high chronic disease risk. Other strengths include a rigorous, internally valid clinical trial design comparing two active interventions with a contact-matched control, and strong initial allocation concealment and treatment fidelity procedures. In addition to objective measurement of MVPA, another strength was a 9 month follow up period allowing examination of both initiation and maintenance of behavior change.

The study also had limitations. Three of four behavioral outcomes were assessed exclusively by self-report and could have been subject to demand characteristics. Fruit and vegetable consumption may have been overestimated and time spent in leisure sedentary screen time underestimated. However, although some risk of self-report bias persists, the objective measure of MVPA derived from the accelerometer also showed large, sustained improvement following the active interventions. Fruit and vegetable intake and sedentary leisure showed the largest improvements in this study, as they had in our prior MBC1 study. Notably, in the MBC1 study, improvements in these two behaviors were also unique in being accompanied by increased self-efficacy, suggesting that changing them is both feasible and empowering [60]. Although intervention benefits persisted through nine months, longer duration follow-up remains needed. A lack of sustained superiority of sequential over simultaneous treatment could have been caused by the fact that goal progression was time-dependent, rather than mastery-based. The sequential intervention added a physical activity goal at week 7, regardless of whether participants had achieved mastery of their fruit and vegetable or sedentary leisure screen time goals. It remains possible that sequential treatment could have increased MVPA even more if the addition of this new target goal had been delayed until initial behavior targets were reached.

Treating multiple risk behaviors simultaneously is inherently more efficient than treating them sequentially, but simultaneous change might be more feasible for some population subgroups than others. In MBC2’s diverse, moderately well-educated study sample, we saw no detrimental effects of intervening on multiple diet and activity behaviors all at once, rather than one at a time. However, in a different trial, people with a greater number of risk behaviors were more likely to drop out when treated simultaneously rather than sequentially [38], an effect that could reflect the association between multiplicity of risk behaviors and social disadvantage [61,62]. If overzealous intervention on too many risk behaviors at once disproportionately drives off marginalized, resource-poor subgroups, there is risk that preventive intervention will fail to reach those in greatest need of help. Caution remains warranted before inferring that all subpopulations and contexts can accomplish unlimited behavior changes at once because: 1) simultaneous (vs sequential) intervention has yielded higher relapse and dropout in some trials; 2) the number of behaviors that can be changed at once remains unknown; and 3) some risk behaviors may be disproportionately hard to change concurrently with others [39,56,63-67].

The finding that an integrated multicomponent connective mHealth intervention produced large, sustained changes in multiple diet and activity behaviors over time is encouraging. Smartphones and wearable sensors are becoming increasingly ubiquitous, equipping consumers with real-world tools that have the potential to support healthy lifestyle changes. Interventions like the present one that combine patient facing technology with digitally connected, personalized coaching support have shown promise [36,68,69]. Including behavior change coaching as a service provided by trained paraprofessionals or artificially intelligent agents could soon make technology-supported behavioral interventions a scalable part of the health care system.

A mobile health intervention integrating a smartphone app, accelerometer, modest initial performance incentives, and remote connected coaching can produce large sustained improvements in multiple prevalent diet and activity risk behaviors, whether physical activity is targeted simultaneously with or sequentially after other risk behaviors.

## Appendix

Multimedia Appendix 1 CONSORT-EHEALTH checklist (V 1.6.1).

Multimedia Appendix 2 Published clinical trial protocol.

## Abbreviations

ANOVA: analysis of variance
MBC1: Make Better Choices 1
MBC2: Make Better Choices 2
MVPA: moderate to vigorous physical activity
SUDS: Subjective Units of Distress Scale
