# Supplementary material for: Multicomponent mHealth Intervention for Large, Sustained Change in Multiple Diet and Activity Risk Behaviors: The Make Better Choices 2 Randomized Controlled Trial
Source: J Med Internet Res. 2018 Jun 19;20(6):e10528. doi: 10.2196/10528 (PMC6030572; doi:10.2196/10528)
Supplement: Multimedia Appendix 2 [file jmir_v20i6e10528_app2.pdf]

Contemporary Clinical Trials 41 (2015) 85–92

Contents lists available at ScienceDirect

Contemporary Clinical Trials

journal homepage: www.elsevier.com/locate/conclintrial

Design and protocol of a randomized multiple behavior change trial: Make Better Choices 2 (MBC2)

Christine A. Pellegrini [a,*], Jeremy Steglitz [a], Winter Johnston [a], Jennifer Warnick [a], Tiara Adams [a], H.G. McFadden [a], Juned Siddique [a], Donald Hedeker [b], Bonnie Spring [a]

[a] Department of Preventive Medicine, Northwestern University Feinberg School of Medicine, 680 N. Lake Shore Drive, Suite 1400, Chicago, IL 60611, United States
[b] Department of Health Studies, University of Chicago, 5841 South Maryland Ave (MC2007), Chicago, IL 60637, United States

ARTICLE INFO

Article history:
Received 21 October 2014
Received in revised form 14 January 2015
Accepted 17 January 2015
Available online 24 January 2015

Keywords:
mHealth
Multiple behavior change
Physical activity
Diet

ABSTRACT

Background: Suboptimal diet and inactive lifestyle are among the most prevalent preventable causes of premature death. Interventions that target multiple behaviors are potentially efficient; however the optimal way to initiate and maintain multiple health behavior changes is unknown.
Objective: The Make Better Choices 2 (MBC2) trial aims to examine whether sustained healthful diet and activity change are best achieved by targeting diet and activity behaviors simultaneously or sequentially.
Study design approximately 250 inactive adults with poor quality diet will be randomized to 3 conditions examining the best way to prescribe healthy diet and activity change. The 3 intervention conditions prescribe: 1) an increase in fruit and vegetable consumption (F/V+), decrease in sedentary leisure screen time (Sed−), and increase in physical activity (PA+) simultaneously (Simultaneous); 2) F/V+ and Sed− first, and then sequentially add PA+ (Sequential); or 3) Stress Management Control that addresses stress, relaxation, and sleep. All participants will receive a smartphone application to self-monitor behaviors and regular coaching calls to help facilitate behavior change during the 9 month intervention. Healthy lifestyle change in fruit/vegetable and saturated fat intakes, sedentary leisure screen time, and physical activity will be assessed at 3, 6, and 9 months.
Significance: MBC2 is a randomized m-Health intervention examining methods to maximize initiation and maintenance of multiple healthful behavior changes. Results from this trial will provide insight about an optimal technology supported approach to promote improvement in diet and physical activity.

© 2015 Elsevier Inc. All rights reserved.

1. Introduction

Two of the most common preventable causes of death in the United States are poor quality diet and physical inactivity [1,2]. In particular, four specific diet and activity behaviors have been linked to increased risk of chronic conditions such as cardiovascular disease, stroke, and cancers. These behaviors are: (1) high saturated fat intake [3], (2) low fruit and vegetable consumption [4,5], (3) insufficient physical activity [6–8], and (4) high sedentary time [9–11].

Research has indicated that changes in lifestyle behaviors can reduce the risk of chronic disease [12,13] and premature death [14]. Unhealthy lifestyle behaviors cluster, such that most adults engage in more than one [15,16]. However, non-adherence to treatment recommendations is problematic when intervening on multiple behaviors [17]. The Make Better Choices (MBC) Study [18] is one example of a successful intervention that initiated and maintained changes in multiple

Abbreviations: MBC2, Make Better Choices 2 study; F/V, Fruit and vegetable; Sed, Sedentary behavior; PA, Physical activity.

* Corresponding author at: Department of Preventive Medicine, Northwestern University, 680 N. Lakeshore Drive, Suite 1400, Chicago, IL 60611, United States. Tel.: +1 312 503 1395; fax: +1 312 908 9588.
E-mail address: c-pellegrini@northwestern.edu (C.A. Pellegrini).

http://dx.doi.org/10.1016/j.cct.2015.01.009
1551-7144/© 2015 Elsevier Inc. All rights reserved.

behaviors among adults who met all four risk behaviors named above. Specifically, participants were randomized to 1 of the 4 conditions involving one dietary change (increase fruit and vegetable consumption [F/V+] or decrease saturated fat) and one activity change (increase physical activity [PA+] or decrease sedentary leisure screen time [Sed−]). Participants were incentivized to meet study goals and received remote coaching supported by a personal digital assistant (PDA). Following a 3 week treatment period, the F/V+, Sed− condition resulted in the greatest improvement in diet and activity behaviors, as compared to the other 3 conditions, yielding healthful changes in fruits and vegetables, sedentary behavior, and saturated fat,. As important, significant improvement was sustained through the 20-week follow-up period [19].

The behavior that displayed the least improvement over the intervention period was physical activity [19]. Consequently, the Make Better Choices 2 (MBC2) trial was developed to determine how to add a significant, sustained improvement in physical activity to the improvements that MBC1's F/V+ and Sed− intervention produced in fruits and vegetables, saturated fat, and sedentary time. Specifically, MBC2 aims to determine whether healthy change in all four diet and activity risk behaviors can be achieved by prescribing PA+ simultaneously with F/V+, Sed− (Simultaneous) or whether greater change in all four risk behaviors is achieved by prescribing F/V+ and Sed− first, followed by PA+ (Sequential). Understanding the mechanisms that guide successful multiple risk behavior change will inform the development of more cost-effective interventions that can target multiple behaviors efficiently.

2. Methods

2.1. Study design

The MBC2 trial is a 3-group prospective randomized controlled trial (RCT) comparing the effects of the three lifestyle intervention conditions: (1) Simultaneously targeting F/V+, Sed−, and PA+ (Simultaneous); (2) Sequentially targeting F/V+, Sed−, first, followed by PA+ (Sequential); (3) Stress management contact control (Control). Outcomes will be assessed at 3 time points: baseline, 3, and 9 months.

The primary outcome of the MBC2 trial is standardized healthy lifestyle change in the four risk behaviors: fruit/vegetable intake, saturated fat intake, sedentary leisure screen time, and physical activity. The secondary outcomes are the behavior change mechanisms: habit strength (automaticity) and superordinate healthy lifestyle goal strength. Exploratory outcomes of the MBC2 trial are change in blood pressure, lipids, and insulin.

2.2. Theoretical framework

The MBC2 design examines two contrasting hypotheses based on theories of multiple behavior change: the Mastery Hypothesis and the Synergy Hypothesis. The Mastery Hypothesis, based on theories of limited self-regulatory strength, posits that the human capacity to intentionally exert self-control via conscious, effortful type 2 processes [20,21] is limited. Attempting to attain multiple behavioral goals is difficult because the self-regulatory resource must be split across directed pursuit of the several behavioral goals being sought. However, as self-regulation becomes more automatic or habitual (i.e., nonconscious) via type 1 processes, less of the self-regulatory resource is required [22–24]. Therefore, the Mastery Hypothesis supports a sequential approach. By initially focusing on improving diet and decreasing sedentary leisure screen time, the sequential approach enables these behaviors to become automatic habits that lack substantial drain on self-regulatory resources. Once these behaviors are mastered and automated, the goal of increasing physical activity can be added since there will be enough self-regulatory resources to devote to attaining an additional goal.

The Synergy Hypothesis, grounded in goal systems theory [25,26], posits that goals are organized in associative cognitive networks [27,28]. Within an associate cognitive network, superordinate goals (e.g., being healthier) are linked to subgoals (e.g., F/V+, Sed−, and PA+). Pursuing a subgoal can spread activation to implicitly prime a superordinate goal or vice versa [29–32]. Further, as additional associated subgoals are pursued, the superordinate goal becomes more strongly activated. The Synergy Hypothesis supports a simultaneous approach to multiple behavior changes such that concurrently assigning several behavior change subgoals will increase the strength of the superordinate goal to be healthier. Moreover, once the superordinate goal has been strongly activated, there is a higher commitment to each of the subgoals.

2.3. Eligibility criteria

Participants must be between 18–65 years old, reside in the Greater Chicagoland area, and be willing to monitor lifestyle behaviors on a smartphone application for 9 months. Participants must also meet the following criteria: 1) consume <5 servings of fruits and vegetables per day; 2) consume ≥8% of daily calories from saturated fat; 3) engage in <150 min of moderate to vigorous intensity physical activity per week; and 4) engage in >120 min/week of sedentary leisure screen time. Participants will be excluded if they have any unstable medical condition (uncontrolled hypertension, diabetes, recent myocardial infarction), use an assistive device for mobility, weigh >350 lbs, have been hospitalized for a psychiatric disorder in the past 5 years, or have any contraindications for exercise. Additional exclusion criteria include: pregnant or lactating, meet criteria for anorexia, bulimia, or binge eating disorder, report suicidal ideation, or indicate substance abuse or dependence (besides nicotine). Individuals who indicate the presence of a stable medical condition such as non-insulin dependent diabetes or hypertension will be required to obtain physician approval to participate.

2.4. Recruitment

Participants will be recruited through the following means: 1) flyers, 2) advertisements on local transportation, 3) brochures disseminated at health fairs and local physicians' offices, 4) online postings, 5) study website, 6) newspaper advertisements, and 7) research registries. Interested participants will be directed to the study website for more information and to complete initial web screening.

2.5. Screening and baseline run-in process

Potential participants will complete a web screener to assess initial eligibility criteria. If eligible, candidates will be contacted by telephone to provide additional study information and assess additional eligibility criteria. After completing telephone screening, participants will be notified of eligibility status and whether medical clearance to participate is necessary. All eligible candidates will be invited to an in-person session.

At the in-person session, potential participants will learn complete study details, ask any questions they may have about participation, and if interested, complete the informed consent process (approved by the Northwestern University Institutional Review Board). After consenting, participants will complete in-person screening. First, physical measures (height, weight, blood pressure, and waist circumference [in duplicate]) will be obtained. Next, HbA1C will be collected using a finger stick method. Staff will also complete brief modified screening interviews to rule out major depression, binge eating, alcohol and substance abuse [33,34]. If eligible, potential participants will receive brief training on how to estimate food portion size, as well as how to use the study equipment to track the five targeted behaviors: diet, physical activity, stress, sleep, and sedentary leisure screen time.

Eligible participants will be loaned a study smartphone and accelerometer to begin baseline recording. Participants will wear the accelerometer daily and use the study native application to self-monitor their behaviors for 7–10 days. Specifically, participants will be asked to record their level of stress three times daily, total sleep hours/day, total sedentary leisure screen time, and all foods and drinks consumed except water. Study candidates' self-monitoring data will be assessed for eligibility at the end of the baseline period, to ensure that participants are willing to self-monitor and exhibit all four risk behaviors.

2.6. Randomization

Prior to randomization, study staff will complete a brief equipoise induction to make salient the pros and cons of being in any of the three conditions. Participants will not be randomized if they express an unwillingness to participate in any condition or a strong desire for one condition. Following the interview, eligible participants will be randomized to one of three intervention conditions: Sequential, Simultaneous, and Control. Randomization assignments, stratified by gender, will be computer-generated using randomly permuted blocks. Participants will learn their random assignment by downloading a condition specific smartphone application.

2.7. Intervention

Active intervention will occur during weeks 1–12 and follow-up will happen during weeks 13 and 40. The intervention for each condition will occur in two phases: prescription and maintenance. During the prescription phase, participants will work to incrementally close the gap between their current behavior, as measured during the baseline recording, and the eventual goals. In the maintenance phase, participants will be encouraged to maintain the behaviors and goals established at the end of the prescription phase. The remaining weeks between 13 and 40 will comprise a follow-up period. Table 1 illustrates the timeline, intervention phases, and goals of the 3 treatment conditions. Study participants will be incentivized $5 per week to meet study goals during the 12-week prescription phase.

Once randomized, participants will receive a smartphone application to self-monitor the specific behaviors targeted in their intervention condition (Fig. 1). For example, the application for the simultaneous condition depicts and helps participants to monitor fruit and vegetable intake, physical activity, and sedentary leisure screen time, whereas the stress management control condition helps participants monitor sleep, stress, and relaxation. Each application provides a visual depiction of both the current state of behavioral accomplishment and the goal. Participants will be encouraged to self-monitor throughout the entire 9 month study.

Participants will receive a binder with study materials specific to their intervention condition. A topic relevant to the behaviors being targeted will be reviewed on each call (Table 2). In addition to discussing the topics on coaching calls, coaches will apply problem solving, motivational interviewing, and goal setting to help participants implement and maintain behavior changes. Coaching calls will occur weekly during the first 12 weeks of participation, bi-weekly during the weeks 13–24, and monthly for the remainder of the 9 month intervention. Calls will last approximately 15 min during the prescription period and 10 min during the follow-up period. All coaching calls will be audio recorded to assess treatment fidelity.

2.7.1. Simultaneous

Participants randomized to the Simultaneous condition will start the 12 week prescription phase by targeting sedentary leisure screen time, fruit and vegetable consumption, and physical activity. Goals will be set during the first 6 weeks to incrementally decrease the discrepancy between baseline and the intervention goal by one third every 2 weeks for each of the 3 behaviors (Table 1). The intervention goals for the behaviors are: 1) ≤90 min/day of sedentary leisure screen time, which includes free time spent sitting and watching TV or movies or making recreational use of a computer, smartphone, or tablet; 2) ≥4.5 cups of fruit and vegetables; and 3) ≥150 min/week of moderate to vigorous physical activity (MVPA). During the maintenance phase (weeks 7–12) of the prescription period, participants will continue working to attain or maintain the end of intervention goals.

Participants will receive the Simultaneous MBC2 smartphone application (Fig. 1a) and an accelerometer to use during the 9 month study. The accelerometer is worn around the waist and participants will be instructed to wear it during waking hours. Physical activity data is transmitted automatically via Bluetooth to the smartphone application, which displays current MVPA as compared to the daily and weekly goals. If participants engage in activity during which the accelerometer cannot be worn (i.e. swimming), they can manually record minutes of MVPA in the app. Participants will also self-monitor dietary intake and minutes of sedentary leisure time throughout the study. The application displays the number of fruits and vegetables consumed and sedentary time accumulated relative to goals.

Table 1
MBC2 intervention by treatment condition.

MBC2 call schedule									
Study week	Simultaneous			Sequential			Stress control		
	Call	FV/SED	Exercise	Call	FV/SED	Exercise	Call	Stress management	Sleep
1	1	To 1/3 FV, SED goals	1/3 to PA	1	To 1/3 FV, SED goals		1	To 1/3 relaxation goals	1/3 To sleep goal
2	2			2			2		
3	3	To 2/3 FV, SED goals	2/3 to PA	3	To 2/3 FV, SED goals		3	To 2/3 relaxation goals	2/3 To sleep goal
4	4			4			4		
5	5	To 3/3 FV, SED goals	3/3 to PA	5	To 3/3 FV, SED goals		5	To 3/3 relaxation goals	3/3 To sleep goal
6	6			6			6		
7	7	Maintain goals		7	Maintain goals	to 1/3 PA goal	7	Maintain goals	
8	8			8			8		
9	9	Maintain goals		9	Maintain goals	to 2/3 PA goal	9	Maintain goals	
10	10			10			10		
11	11	Maintain goals		11	Maintain goals	to 3/3 PA goal	11	Maintain goals	
12	12			12			12		
14	13	Follow up		13	Follow up		13	Follow up	
16	14			14			14		
18	15			15			15		
20	16			16			16		
22	17			17			17		
24	18			18			18		
26	19			19			19		
28	20			20			20		
32	21			21			21		
36	22			22			22		
40	23			23			23		
End goals		4.5 cups fruits and vegetables/day<br>150 min/week physical activity<br><90 min/day sedentary leisure			4.5 cups fruits and vegetables/day<br>150 min/week physical activity<br><90 min/day sedentary leisure			7.5 h of sleep/night<br>3 relaxation exercises/day<br>30% reduction in stress	

2.7.2. Sequential

Participants randomized to the Sequential condition will only aim to modify sedentary leisure screen time and fruit and vegetable intake during the first 6 weeks of the intervention. Similar to the Simultaneous condition, goals will be set to decrease the discrepancy between baseline behavior and the intervention goal incrementally by one third every 2 weeks (Table 1). During the 7th week of the study, Sequential participants will be asked to maintain behavior at the end goal levels for fruits and vegetables and sedentary leisure screen time. They will also begin to increase physical activity and will wear the accelerometer for the remainder of the study. The goal for physical activity will progressively increase by one third every 2 weeks until the end goal of 150 min/week of MVPA is reached.

Sequential participants will receive a smartphone application similar to the one for participants in the Simultaneous condition, except that the physical activity component will not

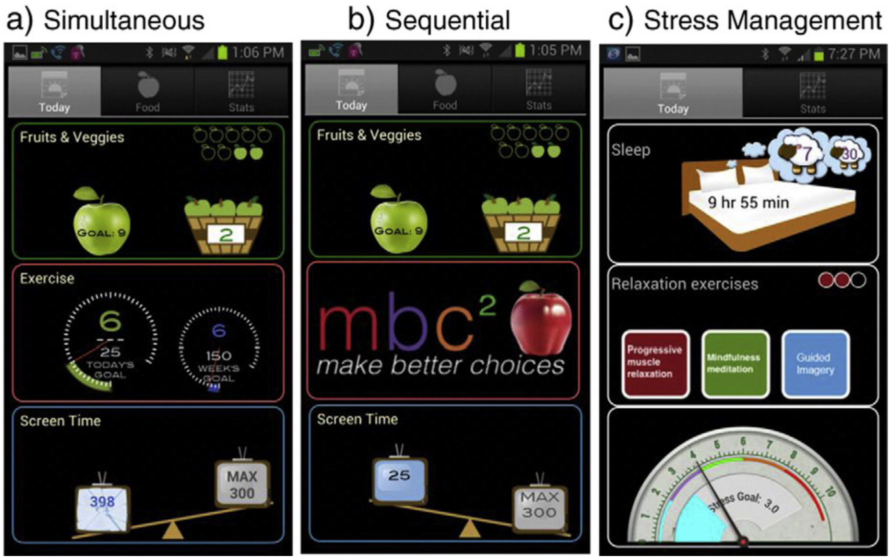

Fig. 1. MBC2 smartphone applications.

Table 2
Session topics by condition.

Simultaneous and sequential		Stress management
Week	Topic	Topic
1	Barriers to change	Progressive muscle relaxation
2	Behavior chains and problem solving	Sleep education
3	Eating out	Pleasant activities
4	Stimulus control	Mini relaxation exercises
5	Grocery shopping	Barriers to change
6	Managing holidays and parties	Behavior chains and problem solving
7	Being active	Mindfulness relaxation
8	Negative thoughts	Negative thoughts
9	Time management	Time management
10	Staying motivated	Staying motivated
11	Relapse prevention	Relapse prevention
12	Action plan for maintenance	Action plan for maintenance

be available during the first 6 weeks of the intervention (Fig. 1b). When participants are given a physical activity goal at the beginning of the 7th week, the application will switch to the one used by Simultaneous participants that allows monitoring of all 3 targeted behaviors.

2.7.3. Stress management

Participants randomized to the Stress Management Control condition will be coached to improve their stress, relaxation, and sleep throughout the 9 month study. In addition, they will be encouraged to complete 3 relaxation exercises each day. The prescribed relaxation exercises include guided imagery and progressive muscle relaxation sessions that are 2, 12, or 20 min in duration.

They will receive a smartphone application specific to their intervention condition (Fig. 1c) and will be asked to monitor the number of hours slept daily and record real-time stress levels at least three times daily. During weeks 1–6, participants' goals will be to progressively reduce the discrepancy between their baseline stress and sleep levels to the end goals of ≥7.5 h of sleep per day and a 30% reduction in stress level. They will rate stress on a scale from 0 (no stress) to 10 (high stress) [35] and will also record daily sleep duration (hours) and quality (1 = poor – 10 = great). Participants will be encouraged to attain and maintain these end goals during the remainder of the intervention period (weeks 7–12) and follow-up (weeks 13–40).

2.8. Fidelity

All randomization and telephone coaching sessions will be audiotaped and a randomly chosen 10% sample will be assessed quarterly by blinded raters in order to determine intervention fidelity. Checklist items will be coded to characterize both the coach's delivery of required elements for an intervention condition (e.g., encouraging a person in the control condition to go to bed earlier) as well as the omission of intervention elements from an alternative intervention condition (e.g., not encouraging a participant in the initial 6 weeks of the sequential condition to get more and better quality sleep or be more physically active). Coaches who do not meet fidelity criteria, defined as scoring 90% or better on fidelity checklist ratings, will be retrained and reevaluated prior to making any additional coaching calls.

2.9. Outcomes

2.9.1. Primary outcomes

The primary outcome is healthy diet and activity improvement aggregated across changes fruit and vegetable intake, saturated fat intake, sedentary leisure screen time, and physical activity. Behaviors will be assessed using the participant's smartphone application self-monitoring data recorded during the baseline, 3, 6, and 9 month assessment periods. During assessment periods, participants in all conditions will self-monitor all behavioral outcomes: dietary intake, sedentary leisure screen time, physical activity, stress level and sleep. Fruit and vegetable and saturated fat intakes will be derived from dietary intake data recorded on the smartphone application. In addition, the Block Food Frequency Questionnaire (FFQ) [36] will be administered at baseline, 3, and 9 months. Sedentary leisure screen time also will be self-monitored in the smartphone application. Although accelerometers will be worn, accelerometry cannot discriminate between discretionary leisure and non-leisure sedentary time; hence, sedentary leisure time will be self-reported. MVPA will be objectively collected via the accelerometer (Shimmer, Dublin, Ireland) and transmitted via Bluetooth to the study smartphone application. The time spent in MVPA will be the primary measure of physical activity.

2.9.2. Secondary outcomes

Two behavior change mechanisms will be examined at each time point: habit strength (automaticity) and superordinate healthy lifestyle goal strength. The 12-Item Self-Reported Habit Index [24] for F/V+, Sed−, and PA+ will assess the degree to which these behavioral improvements have become habitual. Participants will use a 7-point Likert scale (1 = fully disagree; 7 = fully agree) to rate their agreement with statements about the degree to which each behavior has become a habit. The Habit Strength Index exhibits high internal reliability [24] and the 5-item version will be analyzed [37,38]. Superordinate healthy lifestyle goal commitment will be assessed using a modified version of a 9-item measure developed by Hollenbeck et al. [39]. Participants will use 7-point Likert scales (1 = not at all; 7 = very true) to rate their agreement with 4 statements about their commitment and willingness to invest effort in a goal to become healthy.

2.9.3. Exploratory outcomes

At baseline, 3, and 9 months, blood pressure will be assessed using the Omron Hem-907XL (Omron Healthcare, Inc., Bannockburn, IL). . Blood pressure will be measured 3 times at each visit and the average of the 2nd and 3rd reading will be used. Body weight also will be measured using a calibrated balance beam scale with participants wearing light weight clothing and no shoes. Weight will be collected to the nearest 0.25 lb at baseline, 3, and 9 months. Lipids and insulin will be assessed from blood samples taken via antecubital venipuncture and assayed for a lipid panel (total cholesterol, triglycerides, HDL-C, LDL-C) and insulin.

2.10. Statistical analyses

The primary aims of the study are to compare intervention groups for dietary and activity change throughout the study time points. Our basic modeling approach will be to use linear mixed models for longitudinal data [40]. This class of models does not assume that subjects are measured at all time points, and therefore can include subjects with missing data across time. In these analyses, we will examine primarily linear and curvilinear changes across time using orthogonal polynomial transforms, in order to assess the independent effects of these two types of trends. In addition to the effect of time, the main independent variable is group, a between-subjects factor with 3 levels: control, simultaneous, and sequential. Here, we will use a-priori Helmert contrasts [41] to specifically test the following two comparisons; H1: the combined active treatment groups vs. the control group, and H2: simultaneous vs. sequential. We will examine specifically the group by time interaction to test whether healthy lifestyle change across time differs by group, in terms of these two Helmert contrasts.

For the dependent variable(s), to quantify lifestyle behaviors on a common metric, we will generate z-scores reflecting each participant's average daily fruit/vegetable intake, saturated fat intake, daily physical activity, and daily targeted sedentary activity relative to the sample distribution for each of these variables at baseline. By using the baseline means and standard deviations to create these z-scores, we will be able to readily identify change relative to baseline. Z-scores will be standardized such that positive values indicate more healthful eating (higher FV intake, lower saturated fat intake) and more active lifestyle (higher PA, lower targeted sedentary behavior). As in our past study, we will treat as the primary dependent variable the composite of the four z-scores, which is obtained by simply taking the mean of the four z-scores together at a particular time point. This yields a measure of demonstrated meaning that combines information from the four targeted behaviors.

The results of the linear mixed model do depend on the variance–covariance structure that is used in the analysis. In particular, the standard errors for the fixed effects (group, time, group by time) depend on a great extent on the variance–covariance structure. To ensure that our results are reasonable, we will examine several possible structures including models with multiple random effects (e.g., intercept, linear trend, quadratic trend) as well as inclusion of autocorrelated error structures (e.g., AR1, MA1, Toeplitz). We will choose the most reasonable structure using likelihood ratio tests for nested models and Akaike Information Criterion (AIC) for non-nested models. This will ensure that the standard errors, and thus the p-values, for the fixed effects are reasonable.

In addition to the analysis of the composite z-score across time, we will examine each of the four targeted behaviors across time in a similar way. For this, we will use a multivariate linear mixed model to jointly examine changes due to time, group, and group by time for the four targeted behaviors (FV intake, saturated fat intake, PA, sedentary behavior) following the approach detailed in Thiébaut et. al. [42] In doing this, we will follow the same modeling approach as detailed above for the composite z-score using orthogonal polynomials for time and Helmert contrasts for group. Again, our primary interest will be on the tests of the group by time interaction, here, for each of the four behaviors.

2.11. Sample size and power

In terms of power for longitudinal designs, we have used formulas provided in Hedeker et al. [43] for comparing groups in terms of their composite z-scores across time. In the present case, based on the power calculations below, there will be 50 control subjects and 100 subjects in each of the two intervention groups at baseline. Based on our current study, we conservatively assume an attrition rate of 20% at the final 9-month timepoint (i.e., there will be at least 200 subjects at the final timepoint). Also, based on an estimate from our current study, we assume that the intraclass correlation of the composite z-scores across time equals 0.5. Our primary aim consists of comparing (a) the two active treatment groups (simultaneous and sequential) combined to the control group, and (b) comparing simultaneous vs. sequential. These correspond to Helmert contrasts for the group factor [41]. Below, we present power calculations for each of these comparisons. To establish the effect size that we wanted to base the study's power on, we examined the data from our previous study [19], specifically comparing the MBC group (FV + Sed −) to the intervention arm that was least successful (Fat − PA +). It should be noted that this latter arm did have a positive effect on the composite z-score over time (but was not as successful as the FV + Sed − group). Using this “control” group, the average effect size (mean difference in composite z-score divided by common standard deviation) equaled 0.46. This is averaged across the post-baseline follow-up time points, but the individual time point effect sizes were not appreciably different from this. We believe that the first Helmert contrast will be larger than this effect size, but that the second, comparing the two MBC groups to each other, might be a bit smaller than this. Based on this information, we decided to power the study for an effect size in the range of 0.5 for the first Helmert contrast (H1: Simultaneous + Sequential versus control) and 0.4 for the second Helmert contrast (H2: Simultaneous vs. Sequential).

Based on the above assumptions, the effect sizes listed below can be detected with power greater than .80 or .90 for a two-tailed 0.05 hypothesis test. Note, that we have calculated power for both a common group difference across all post-baseline time points (same effect size at the post-baseline time points) and an emerging group difference across time (zero effect size at baseline that grows to the indicated effect size at the final 9-month time point). We primarily expect the former, but will also test for the latter possibility.

	Common group difference		Emerging group difference	
Contrast	Power = 0.80	Power = 0.90	Power = 0.80	Power = 0.90
H1	0.37	0.43	.40	0.46
H2	0.33	0.38	.36	0.41

As can be seen, all of these detectable effect sizes are equal to or below the observed effect size of 0.46 based on our prior study. Also, all of the H1 effect sizes are below the 0.5 level and all of the H2 effect sizes are near or below the 0.4 level.

3. Discussion

Unhealthful behaviors, such as poor quality diet and physical inactivity, have been found to cluster together, augmenting the negative effect on health outcomes [44]. The risk for various chronic diseases increases with the number of unhealthy risk factors present [45,46], and medical costs also rise. One estimate is that each risk factor adds an additional $350 in medical cost burden, such that behavioral factors account for approximately 21–31% of medical care costs [47]. Capitalizing upon a teachable moment to intervene upon several health behaviors, rather than just one, provides an opportunity to improve healthy lifestyle efficiently and cost-effectively.

Even though poor quality diet and physical inactivity often cluster, the mechanisms and the treatment implications of their covariation remain poorly understood. In previous studies, varying the number and type of behavioral targets and the timing and sequencing of changes has yielded mixed results [48–52]. The ideal number of behaviors to target for change, as well as the timing of when to target the behaviors remain unknown. Also unclear is how the increasing use of mobile technologies as a scaffolding to deliver interventions influences the optimal configuration of treatment components. Thus, the current MBC2 trial aims to examine whether prescribing simultaneous or sequential changes in diet and physical activity with the assistance of a smartphone application results in greater adoption and maintenance of healthy lifestyle improvements over a 9 month period.

A limitation of the MBC2 trial is that participants may be at higher risk than the general population since they must exhibit all four diet and activity risk behaviors in order to be eligible for study participation. Also, sustainability will be limited by the protocol that all participants will be provided with a smartphone and service to use throughout the study at no personal cost. Provision of a smartphone was still considered necessary for this trial, as only approximately 58% of adults own a smartphone [53]. Since smartphone ownership is increasing rapidly in the population, we believe that in the near future, individuals will be able to use their own personal devices to access Make Better Choices 2 and other similar interventions. The growing penetration of mobile technologies can therefore help to increase the reach and accessibility of behavioral interventions to improve public health.

Results from the Make Better Choices 2 (MBC2) trial will provide a better understanding of whether multiple diet and activity behaviors are optimally addressed simultaneously or sequentially. This knowledge will better inform the development of m-Health interventions to foster the initiation and maintenance of healthy diet and physical activity change.

Acknowledgments

Supported by NIH R01 grant HL075451-09 to Dr. Spring.

References

[1] Danaei G, et al. The preventable causes of death in the United States: comparative risk assessment of dietary, lifestyle, and metabolic risk factors. PLoS Med 2009;6(4):e1000058.
[2] Mokdad AH, et al. Actual causes of death in the United States, 2000. JAMA 2004;291(10):1238–45.
[3] Sieri S, et al. Dietary fat intake and development of specific breast cancer subtypes. J Natl Cancer Inst 2014;106(5):dju068.
[4] Riboli E, Norat T. Epidemiologic evidence of the protective effect of fruit and vegetables on cancer risk. Am J Clin Nutr 2003;78(3 Suppl.): 559S–69S.
[5] He FJ, et al. Increased consumption of fruit and vegetables is related to a reduced risk of coronary heart disease: meta-analysis of cohort studies. J Hum Hypertens 2007;21(9):717–28.
[6] Kohl HW. Physical activity and cardiovascular disease: evidence for a dose response. Med Sci Sports Exerc 2001;33(6 Suppl.):S472–83 [discussion S493-4].
[7] Thune I, Furberg AS. Physical activity and cancer risk: dose–response and cancer, all sites and site-specific. Med Sci Sports Exerc 2001;33(6 Suppl.): S530–50 [discussion S609-10].
[8] Lee CD, Folsom AR, Blair SN. Physical activity and stroke risk: a meta-analysis. Stroke 2003;34(10):2475–81.
[9] Chomistek AK, et al. Relationship of sedentary behavior and physical activity to incident cardiovascular disease: results from the Women's Health Initiative. J Am Coll Cardiol 2013;61(23):2346–54.
[10] Grontved A, Hu FB. Television viewing and risk of type 2 diabetes, cardiovascular disease, and all-cause mortality: a meta-analysis. JAMA 2011;305(23):2448–55.
[11] Wilmot EG, et al. Sedentary time in adults and the association with diabetes, cardiovascular disease and death: systematic review and meta-analysis. Diabetologia 2012;55(11):2895–905.
[12] Knowler WC, et al. Reduction in the incidence of type 2 diabetes with lifestyle intervention or metformin. N Engl J Med 2002;346(6):393–403.
[13] Sacks FM, Katan M. Randomized clinical trials on the effects of dietary fat and carbohydrate on plasma lipoproteins and cardiovascular disease. Am J Med 2002;113(9, Supplement 2):13–24.
[14] Paffenbarger Jr RS, et al. The association of changes in physical-activity level and other lifestyle characteristics with mortality among men. N Engl J Med 1993;328(8):538–45.
[15] Fine LJ, et al. Prevalence of multiple chronic disease risk factors. 2001 National Health Interview Survey. Am J Prev Med 2004;27(2 Suppl.):18–24.
[16] Pronk NP, et al. Meeting recommendations for multiple healthy lifestyle factors. Prevalence, clustering, and predictors among adolescent, adult, and senior health plan members. Am J Prev Med 2004;27(2 Suppl.):25–33.
[17] Middleton KR, Anton SD, Perri MG. Long-term adherence to health behavior change. Am J Lifestyle Med 2013;7(6):395–404.
[18] Spring B, et al. Make Better Choices (MBC): study design of a randomized controlled trial testing optimal technology-supported change in multiple diet and physical activity risk behaviors. BMC Public Health 2010;10:586.
[19] Spring B, et al. Multiple behavior changes in diet and activity: a randomized controlled trial using mobile technology behavior changes in diet and activity. Arch Intern Med 2012;172(10):789–96.
[20] Baumeister RF, Vohs KD, Tice DM. The strength model of self-control. Curr Dir Psychol Sci 2007;16:396–403.
[21] Muraven M, Baumeister RF. Self-regulation and depletion of limited resources: does self-control resemble a muscle? Psychol Bull 2000; 126(2):247–59.
[22] Fitzsimons GM, Bargh JA. Automatic self-regulation. In: Baumeister RF, Vohs KD, editors. Handbook of self-regulation: research, theory, and application. Guildford Press; 2004.
[23] Gollwitzer PM, Fujita K, Otettingen G. Planning and the implementation of goals. In: Baumeister RF, Vohs KD, editors. Handbook of self-regulation: research, theory, and application. Guilford Press; 2004.
[24] Verplanken B. Beyond frequency: habit as mental construct. Br J Soc Psychol 2006;45(Pt 3):639–56.
[25] Kruglanski AW. Goals as knowledge structures. In: Gollwitzer PM, Bargh JA, editors. The psychology of action: linking cognition and motivation to behavior. New York: Guilford Press; 1996.
[26] Kruglanski AW, et al. A theory of goal systems. In: Zanna MP, editor. Advances in experimental social psychology, Vol. 34. San Diego: Academic Press; 2002.
[27] Andersen JR. The architecture of cognition. Cambridge, MA: Harvard University Press; 1983.
[28] Anderson JR, et al. An integrated theory of the mind. Psychol Rev 2004; 111(4):1036–60.
[29] Bargh JA, Gollwitzer PM. Environmental control of goal-directed action: automatic and strategic contingencies between situations and behavior. In: Spaulding WD, editor. Nebraska symposium on motivation: integrative views of motivation, cognition, and emotion, Vol. 41. Lincoln, NE: University of Nebraska Press; 1994.
[30] Chartrand TL, Bargh JA. Automatic activation of impression formation and memorization goals: nonconscious goal priming reproduces effects of explicit task instructions. J Pers Soc Psychol 1996;71:464–78.
[31] Higgins ET. Goal activation: accessibility, applicability, and salience. In: Higgins ET, Kruglanski AW, editors. Social psychology: handbook of basic principles. New York: Guilford Press; 1996.

[32] Shah JY, Kruglanski AW. When opportunity knocks: bottom-up priming of goals by means and its effects on self-regulation. J Pers Soc Psychol 2003; 84:1109–22.
[33] Sheehan DV, et al. The mini-international neuropsychiatric interview (M.I.N.I.): the development and validation of a structured diagnostic psychiatric interview for DSM-IV and ICD-10. J Clin Psychiatry 1998; 59(Suppl. 20):22–33 [quiz 34–57].
[34] Spitzer RL, Kroenke K, Williams JB. Validation and utility of a self-report version of PRIME-MD: the PHQ primary care study. Primary care evaluation of mental disorders. Patient Health Questionnaire. JAMA 1999;282(18):1737–44.
[35] Kaplan DM, Smith T. A validity study of the subjective unit of discomfort (SUD) score. Meas Eval Couns Dev 1995;27(4):195–9.
[36] Molag ML, et al. Design characteristics of food frequency questionnaires in relation to their validity. Am J Epidemiol 2007;166(12):1468–78.
[37] Gardner B, et al. Towards parsimony in habit measurement: testing the convergent and predictive validity of an automaticity subscale of the Self-Report Habit Index. Int J Behav Nutr Phys Act 2012;9:102.
[38] Rhodes RE, de Bruijn GJ. Automatic and motivational correlates of physical activity: does intensity moderate the relationship? Behav Med 2010; 36(2):44–52.
[39] Hollenbeck JR, Williams CR, Klein HJ. An empirical examination of the antecedents of commitment to difficult goals. J Appl Psychol 1989;74(1): 18–23.
[40] Hedeker D, Gibbons RD. Longitudinal data analysis. New York: Wiley; 2006.
[41] Bock RD. Multivariate statistical methods in behavioral research. New York: McGraw Hill; 1975.
[42] Thiebaut R, et al. Bivariate linear mixed models using SAS proc MIXED. Comput Methods Programs Biomed 2002;69(3):249–56.
[43] Hedeker D, Gibbons RD, Waternaux C. Sample size estimation for longitudinal designs with attrition: comparing time-related contrasts between two groups. J Educ Behav Stat 1999;24(1):70–93.
[44] Prochaska JJ, Prochaska JO. A review of multiple health behavior change interventions for primary prevention. Am J Lifestyle Med 2011;5(3): 208–21.
[45] Djoussé L, Driver JA, Gaziano J. Relation between modifiable lifestyle factors and lifetime risk of heart failure. JAMA 2009;302(4):394–400.
[46] Wannamethee SG, Shaper AG, Whincup PH. Modifiable lifestyle factors and the metabolic syndrome in older men: effects of lifestyle changes. J Am Geriatr Soc 2006;54(12):1909–14.
[47] Edington DW. Emerging research: a view from one research center. Am J Health Promot 2001;15(5):341–9.
[48] Hyman DJ, et al. Simultaneous vs sequential counseling for multiple behavior change. Arch Intern Med 2007;167(11):1152–8.
[49] Vandelanotte C, et al. A randomized trial of sequential and simultaneous multiple behavior change interventions for physical activity and fat intake. Prev Med 2008;46(3):232–7.
[50] Spring B, et al. Randomized controlled trial for behavioral smoking and weight control treatment: effect of concurrent versus sequential intervention. J Consult Clin Psychol 2004;72(5):785–96.
[51] Goodpaster BH, et al. Effects of diet and physical activity interventions on weight loss and cardiometabolic risk factors in severely obese adults: a randomized trial. JAMA 2010;304(16):1795–802.
[52] Taylor WC, et al. Readiness to change physical activity and dietary practices and willingness to consult healthcare providers. Health Res Policy Syst 2004;2(1):2.
[53] Smith A. Pew Internet & American Life Project: mobile technology fact sheet. [cited 2014 June 10]; Available from: http://www.pewinternet.org/fact-sheets/mobile-technology-fact-sheet/; 2014.
